# Ranibizumab versus Bevacizumab for Ophthalmic Diseases Related to Neovascularisation: A Meta-Analysis of Randomised Controlled Trials

**DOI:** 10.1371/journal.pone.0101253

**Published:** 2014-07-01

**Authors:** Bin Wu, Haixiang Wu, Xiaoyan Liu, Houwen Lin, Jin Li

**Affiliations:** 1 Department of Pharmacy, Ren Ji Hospital, affiliated with the School of Medicine, Shanghai Jiao tong University, Shanghai, China; 2 Department of Ophthalmology, and Visual Science, Eye, and ENT Hospital, Shanghai Medical College, Fudan University, Shanghai, China; 3 Department of Ophthalmology, Ren Ji Hospital, affiliated with the School of Medicine, Shanghai Jiao tong University, Shanghai, China; Copenhagen University Hospital Roskilde and the University of Copenhagen, Denmark

## Abstract

**Background:**

Bevacizumab is believed to be as effective and safe as ranibizumab for ophthalmic diseases; however, its magnitude of effectiveness and safety profile remain controversial. Thus, a meta-analysis and systematic review appears necessary.

**Methods:**

PubMed and EMBASE were systematically searched with no restrictions. All relevant citations comparing ranibizumab and bevacizumab were considered for inclusion. Pooled effect estimates were obtained using a fixed- and random-effects meta-analysis.

**Results:**

Nine independent randomised-controlled clinical trials (RCTs) involving 2,289 participants were identified. Compared with bevacizumab, the overall combined weighted mean difference (WMD) of the mean change in visual acuity for ranibizumab was 0.52 letters (95% CI −0.11–1.14). The odds ratios (ORs) of gaining ≥15, gaining 5–14, losing 5–14 and losing ≤15 letters were 1.10 (95% CI 0.90–1.33), 0.93 (95% CI 0.77–1.11), 0.89 (95% CI 0.65–1.22) and 0.95 (95% CI 0.73–1.25), respectively. The risk of serious systemic events increased by 17% (95% CI 6%–27%, p = 0.0042) for bevacizumab treatment in comparison with ranibizumab. No statistically significant differences between the two treatments were found for the nonfatal arterial thrombotic events, ocular serious adverse, death from vascular and all causes events.

**Conclusions:**

Bevacizumab is not inferior to ranibizumab as a treatment for achieving visual acuity. The use of bevacizumab was associated with an increased risk of developing serious systemic events. Weighing the costs and health outcomes is necessary when selecting between bevacizumab and ranibizumab for ophthalmic diseases. Due to the limitations of the available data, further research is needed.

## Introduction

Pathological angiogenesis, a process mainly driven by vascular endothelial growth factor (VEGF), is a hallmark of cancer and various ischaemic and inflammatory diseases.[1], [2] For several ophthalmic diseases involving neovascularisation or increased vascular permeability, such as neovascular age-related macular degeneration (AMD), diabetic macular oedema (DME), or diabetic retinopathy, VEGF-A is a critical regulator of ocular angiogenesis and vascular permeability.[3] These discoveries have resulted in the development of antineoplastic agents for reducing pathologic angiogenesis, such as bevacizumab and ranibizumab.[4]

Bevacizumab (Avastin, Genentech Inc., South San Francisco, California), a recombinant humanised monoclonal IgG1 antibody against all isoforms of VEGF-A, is able to block the binding between VEGF and its receptors (Flt-1 and KDR) on the surface of endothelial cells.[5] Bevacizumab has been widely prescribed in the treatment of many types of malignancy, including colorectal cancer, renal cell carcinoma, lung cancer and breast cancer. Due to its size (molecular weight of 150 kDa) and resultant weak penetration through the retinal layers after intravitreal injection, bevacizumab was thought to have limited efficacy in ophthalmic disease. However, it has been widely used outside of its licensed indications, such as for AMD.[6], [7] In contrast to bevacizumab, ranibizumab (Lucentis, Genentech, Inc., South San Francisco, CA) is a 48 kDa antigen-binding fragment (Fab) form of the bevacizumab molecule. Ranibizumab was specifically developed for ocular indications.[8] With its increased potency, enhanced penetration, and lower possibility of complement-mediated or cell-dependent cytotoxicity, ranibizumab has been an effective treatment for neovascular AMD during several pivotal clinical trials.[9], [10] It has been approved for the treatment of patients with neovascular AMD by the Food and Drug Administration and by the European Medicines Agency since 2006 and 2007, respectively.[11]

The interest in securing approval for bevacizumab in treating ophthalmic diseases of neovascularisation is mainly due to the potential cost savings (per-dose cost, approximately $2,000 for ranibizumab and $50 for bevacizumab), despite the resistance of the pharmaceutical companies concerned.[12], [13] To determine whether bevacizumab is as effective and safe as ranibizumab, numerous randomised, controlled clinical trials (RCTs) and retrospective studies have been performed over the past five years, such as the Comparison of Age-related macular degeneration Treatments Trials (CATT), the Alternative treatments to Inhibit VEGF in Age-related choroidal Neovascularization (IVAN), the Multicenter Anti-VEGF Trial in Austria (MANTA), and the Groupe d'Etude Français Avastin versus Lucentis dans la DMLA néovasculaire (GEFAL).[14]–[22] Although the results of these studies indicated the two drugs to be both effective and safe, subtle differences in their comparative efficacy and safety profiles still exist, as suggested by the different trends of improved visual acuity with ranibizumab in the CATT and GEFAL studies, which should be further assessed. Our aim was to compare the clinical effectiveness and safety between ranibizumab and bevacizumab by performing a systematic review of head-to-head RCTs. Furthermore, this information could be used to address the question of whether off-label bevacizumab therapy is as effective and safe as licensed ranibizumab therapy for patients with ophthalmic diseases.

## Methods

### Search Strategy

This analysis was performed according to the preferred reporting items for systematic reviews and meta-analyses (PRISMA) guidelines and the methods described in the Cochrane Handbook.[23] Two investigators independently (HW and XL) searched all eligible studies in four electronic databases, including PubMed and EMBASE (Excerpta Medica Database), until August 2013; no specific restrictions on language or publication year were applied. The electronic search strategy included the terms (“macular degeneration” OR “retinal degeneration” OR “retinal neovascularisation” OR “choroidal neovascularisation” OR “macula Lutea” OR “diabetic retinopathy” OR “macular oedema”) AND (“bevacizumab or avastin” OR “ranibizumab or lucentis or rhufab”) combined with “randomised controlled trial”. The titles and abstracts were scanned to exclude any clearly irrelevant studies. Furthermore, to identify any additional published reports, a manual search was performed by checking all the references of original reports. In addition, the cited lists of eligible studies by Google Scholar were reviewed to ensure that all appropriate studies were included. The results were compared, and any questions or discrepancies were resolved through iteration and consensus.

This study is an analysis of published data and did not require ethics committee approval.

### Inclusion Criteria

To be eligible, studies had to fulfil the following inclusion criteria: (1) comparative RCT study; (2) study population with ophthalmic diseases; (3) head-to-head comparison of results between ranibizumab and bevacizumab; and (4) full text manuscript available. Exclusion criteria were the following: (1) non-RCT; (2) RCTs that enrolled less than 20 patients; (3) patients previously treated with VEGF inhibitors or patients receiving systemic anti-VEGF therapy; (4) less than six months follow-up; and (5) studies without data from a comparison group.

### Outcome Measures

The primary outcome chosen was change from baseline in the best corrected visual acuity (BCVA) (Snellen equivalent) measured on Early Treatment of Diabetic Retinopathy Study (ETDRS) charts after at least six months of follow-up, including the proportions of patients whose acuity increased by ≥15, increased by 5–14, decreased by 5–14, or decreased by ≤15 letters, as well as the mean number of letters.

Secondary measures evaluated included any ocular/systemic adverse events (death from any cause, arteriothrombotic event, or serious ocular event) and assessment of CNV by fluorescein angiography or OCT.

### Data Extraction and Quality Assessment

Articles were reviewed and cross-checked independently by two investigators (HW and XL), and disagreements were resolved by consensus. Data extracted included the following: the first author's last name, year of publication, study design, number of patients, age, specific ocular disease, intervention, injection times, follow-up days (Table 1), efficacy, and adverse event data. The included studies were critically evaluated using the Jadad composite scale, which scores studies' based on their descriptions of randomisation (2 points), blinding (2 points), and attrition information (1 point).[24] If the above data were not available in the published study, the authors were contacted and asked to supply the information.

**Table 1:** Characteristics of randomised controlled trials included in the meta-analysis.

Study	Ocular disease	Patients for analysis		Baseline visual acuity (letters)		Follow-up (months)	Dosage (mg)		Treatment pattern	Injections per patient(mean)	
		Ranibizumab	Bevacizumab	Ranibizumab	Bevacizumab		Ranibizumab	Bevacizumab		Ranibizumab	Bevacizumab
GEFAL,2013[17]	AMD	183	191	55.78±13.99	54.62±14.07	12	0.5	1.25	As needed after the first injection	6.5	6.8
CATT,2012[14]	AMD	134	129	59.9±14.2	60.2±13.6	24	0.5	1.25	Monthly	11.7	11.9
CATT,2012[14]	AMD	264	251	61.6±13.1	60.6±13.0	24	0.5	1.25	As needed	12.6	14.1
IVAN,2013[15]	AMD	314	296	61.8±15	61.1±15.5	24	0.5	1.25	mix of monthly and as needed	18	19
MENTA,2013[16]	AMD	163	154	56.4±13.5	57±13	12	0.5	1.25	monthly for 3 month then as needed	5.8	6.1
MANJU,2010[18]	AMD	15	7	34.9±14.5	34.9±14.5	12	0.5	1.25	monthly for 3 month then as needed	3.9	7.6
MAGDA,2010[19]	PM	16	16	26.44±12.58	29.5±12.98	6	0.5	1.25	as needed after the first injection	2.81	2.44
PIERLUIGI,2012[20]	PM	23	25	29.5±16	30.5±14	18	0.5	1.25	as needed after the first injection	2.56	4.72
MAURIZIO,2013[21]	RAP	24	26	33±16.5	29.5±10.5	12	0.5	1.25	monthly for 3 month then as needed	3.9	4.6
ANTONIO,2013[22]	DME	28	32	31.5±3	30±2.5	12	0.5	1.5	monthly for 3 month then as needed	7.67	9.84

### Statistical Analysis

The pooled odds ratio (OR), weighted mean difference (WMD) and relative risk (RR) with 95% CI between ranibizumab and bevacizumab were used to estimate the effect and ADR sizes using the ‘metafor’ and ‘meta’ packages, respectively, in R version 2.15.1 for Windows (The R Foundation for Statistical Computing, Vienna, Austria). Statistical heterogeneity among the studies was assessed using Cochran's Q test and the I^2^ statistic.[25]. A value of I^2^>50% was considered to indicate substantial heterogeneity, and a P value<0.05 was considered to suggest significant heterogeneity.[26] Fixed effects models were employed if there was low heterogeneity (I^2^<30%); otherwise, random effects models were used. To assess whether publication bias may have impacted the statistical results, a funnel plot was created, and Egger's and Begg's tests were performed.[27], [28] For Egger's and Begg's tests, P<0.05 was considered to be statistically significant. All statistical tests were two-sided.

## Results

### Study Selection

**Figure 1:**
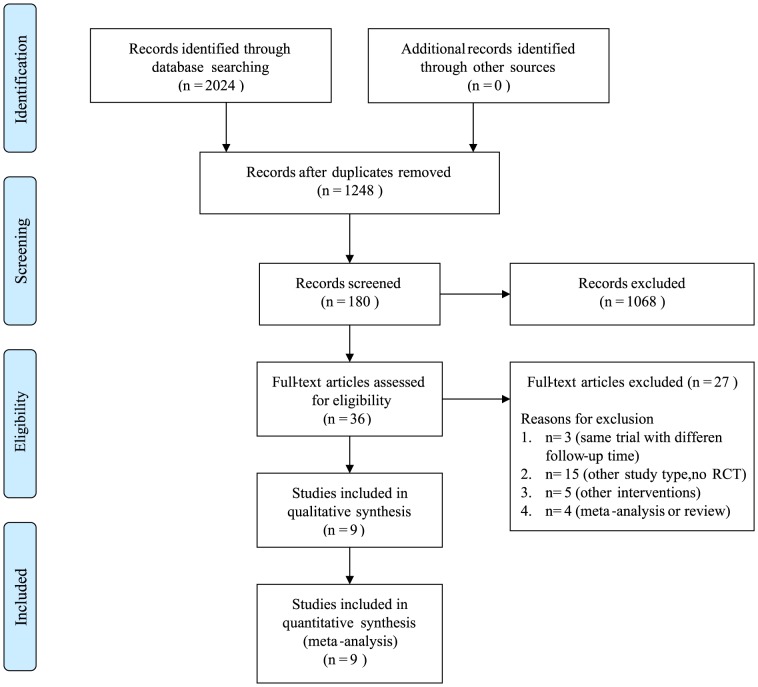
Selection process for randomised controlled trials on the effects of ranibizumab and bevacizumab therapy on ophthalmic diseases.

The literature search produced a total of 2,024 citations, of which 180 were considered potentially relevant (Figure 1). Of these, 37 articles were considered of interest, and their full texts were retrieved for detailed evaluation. Twenty-seven of these 36 articles were subsequently excluded, and the remaining 9 articles were included in the meta-analysis.

### Study Characteristics

Nine independent RCT studies enrolled 2,289 individuals, including 1,162 patients assigned to the ranibizumab arm and 1,127 patients assigned to the bevacizumab arm.[14]–[22] All qualified articles were published between March 2010 and November 2013, and all were in the English language. Five studies focused on AMD, two on pathologic myopia (PM), and one each on retinal angiomatous proliferation (RAP) and diabetic macular oedema (DME). The methodological quality of the included studies was generally good. The average follow-up duration ranged from 6 to 24 months. Patients were followed for an average of over 1 year in a majority of studies (88.9%). The number of included patients in the study varied from 20 to 778, with the two largest studies recruiting over 500 participants each (Table 1).[14], [15]

In all of the studies, the dose was 0.50 mg for ranibizumab. Eight studies administered 1.25 mg for bevacizumab, and only one used 1.5 mg of bevacizumab for DME.[22] The CATT study compared the outcomes of monthly and as needed interventions in four arms.[14] In the IVAN study, outcomes were reported in the ranibizumab and bevacizumab groups with a mixed schedule of monthly and as needed administrations.[15] All other studies had two arms on a monthly schedule for 1 or 3 months, followed by as needed administrations. Change in visual acuity scores from baseline (letters) was available for all studies. Five studies (55.6%) reported data from an adverse event: serious ocular events (five studies), nonfatal arterial thrombotic events (four studies), deaths from any cause (four studies), and deaths from vascular causes (two studies). There were no differences in the baseline age, visual acuity, and foveal thickness between the patients receiving ranibizumab and bevacizumab.

### Effects of interventions

**Figure 2:**
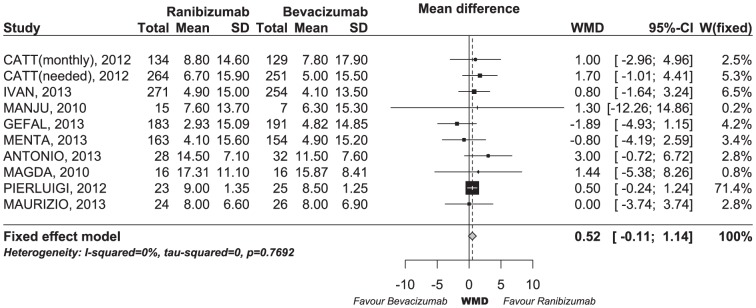
Forest plot of WMD for visual acuity change. CI, confidence interval; WMD, weighted mean difference.

**Figure 3:**
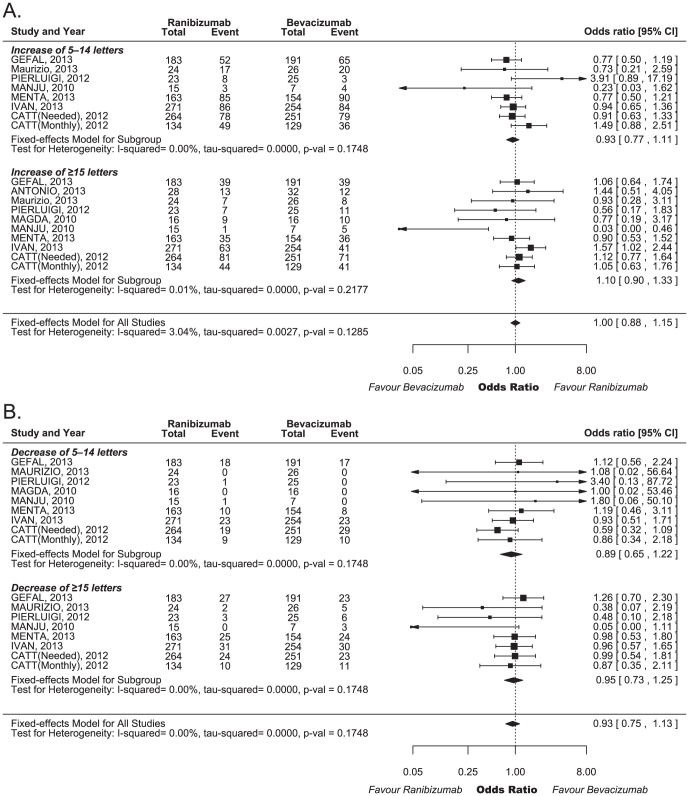
Forest plot of the odds ratios (OR) for visual acuity changes. (A) Gain of ≥15 letters and 5–14 letters; (B) Loss of 5–14 letters or ≥15 letters. CI, confidence interval; OR, odds ratio.

The WMD in the change in the visual acuity score from baseline for ranibizumab versus bevacizumab was 0.52 letters (95% CI −0.11–1.14, p = 0.1046), as shown in Figure 2. Figure 3 shows the results from the fixed-effects model, combining the ORs for the changed proportions of visual acuity scores. Overall, ranibizumab, compared with bevacizumab, did not result in a statistically significant improvement in the visual acuity to the degree of ≥15 (OR: 1.10 [95% CI 0.90–1.33, p = 0.3549]) or 5–14 letters (OR: 0.93 [95% CI 0.77–1.11, p = 0.4206]). The pooled ORs of visual acuity for ranibizumab relative to bevacizumab were 0.89 (95% CI 0.65–1.22, p = 0.4602) for a 5–14 letter decrease and 0.95 (95% CI 0.73–1.25, p = 0.7241) for a ≤15 letters decrease. We did not observe heterogeneity among these studies, despite clear disparities in the ocular disease types and treatment patterns.

**Figure 4:**
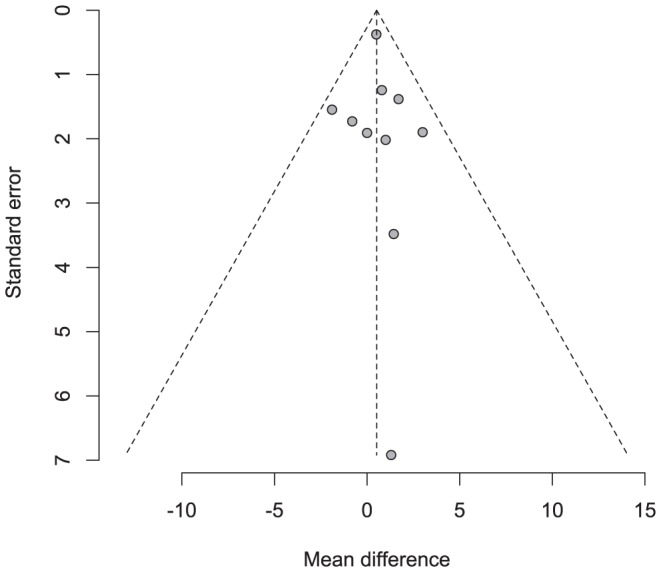
Funnel plot examining the effects of ranibizumab versus bevacizumab therapy.

Neither the funnel plot nor the Egger's and Begg's tests demonstrated any evidence of publication bias for ranibizumab versus bevacizumab (Egger's, p = 0.8365; Begg's, p = 0.5312) (Figure 4).

### Adverse Events

**Figure 5:**
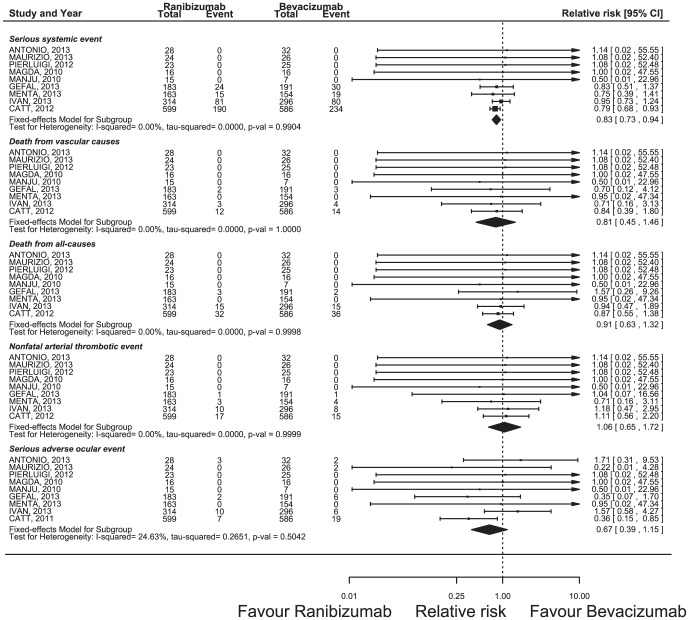
Forest plot of the odds ratios (OR) for adverse events. CI, confidence interval; OR, odds ratio.

We conducted a meta-analysis to estimate the overall RR of ADRs associated with ranibizumab versus bevacizumab. Other than serious systemic events, no statistically significant heterogeneity was found among the studies included in the analysis, despite clear disparities in the ocular disease types and treatment pattern (Figure 5). Using a fixed-effect model, the summary overall RRs for ranibizumab versus bevacizumab were 0.83 (95% CI 0.73–0.94, p = 0.0035) for serious systemic events, 0.81 (95% CI 0.45–1.46, p = 0.4845) for death from vascular causes, 0.91 (95% CI 0.63–1.32, p = 0.6329) for death from all causes, 1.06 (95% CI 0.65–1.72, p = 0.8226) for nonfatal arterial thrombotic events, and 0.67 (95% CI 0.39–1.15, p = 0.1454) for ocular serious adverse events.

## Discussion

This study goes beyond prior analyses regarding the choice between ranibizumab and bevacizumab for the treatment of ophthalmic diseases. Improved confidence in the findings is obvious through the incorporation of results from 9 RCT trials representing nearly 2,300 patients. As such, the findings from this study have implications for treatment and for study interpretation and design.

Our meta-analysis demonstrated the non-inferiority of bevacizumab against ranibizumab by using the strict criteria of a non-inferior margin of 3.5 letters in the IVAN study, although the results trend favoured ranibizumab.[15] Likewise, we observed no significant differences in the specific degrees of visual acuity change, including an increase of ≥15, an increase of 5–14, a decrease of 5–14, and a decrease of ≤15 letters. The findings were consistent with the results of the study reported by Chakravarthy U and colleagues, which showed the WMD of the change between ranibizumab and bevacizumab in visual acuity to be 1.15 (95%CI: −0.51 to 2.82) by pooling IVAN and CATT data.[15] To the best of our knowledge, this meta-analysis is the first to confirm the non-inferiority of bevacizumab relative to ranibizumab for ophthalmic diseases by pooling more than two head-to-head RCT trials. The results of non-RCT and preliminary studies that directly compared the outcomes of the two drugs also indicated the efficacy of bevacizumab to be non-inferior to that of ranibizumab in patients with corneal neovascularisation, wet AMD, glaucoma, polypoidal choroidal vasculopathy, RAP or DME.[29]–[35] Based on an indirect comparison in a systematic review for AMD, Ford JA and colleagues found that 27% of patients treated with bevacizumab and 39% of those treated with ranibizumab experienced an improvement in their best corrected visual acuity of >2 lines [OR: 0.95 (95% credible interval 0.23 to 4.32)], which suggests no difference in the relative effectiveness of bevacizumab and ranibizumab. However, this indirect comparison had wide credibility intervals and was not able to exclude the possibility that either drug might be superior. [36]

The safety and tolerability of anti-VEGF therapy is a concern of clinicians and patients, especially regarding arterial thromboembolic events.[37], [38] One meta-analysis showed that the intravitreal use of anti-VEGF antibodies was not associated with an increased risk of arterial thromboembolic events.[39] Our meta-analysis focused on the relative safety of bevacizumab and ranibizumab. We found that the bevacizumab arm, compared with the ranibizumab arm, experienced a significant 17% increased risk of developing serious systemic events, consistent with the findings reported by Chakravarthy U and colleagues[15]. In a retrospective cohort study of 146,942 Medicare beneficiaries 65 years or older with a claim for AMD, Curtis and colleagues found that the adjusted hazard ratios for ranibizumab versus bevacizumab were 0.86 (95% CI 0.75–0.98) for mortality and 0.78 (95% CI 0.64–0.96) for stroke, respectively.[40] However, in contrast to this finding, other studies have suggested that there is no sufficient evidence to conclude that there is a difference between the safety profiles of the different VEGF inhibitors.[11], [41] Considering these controversies and the differential cost, cheaper bevacizumab treatments may be continued if the benefits of the drug outweigh the risk. Furthermore, the treatment patterns of bevacizumab should be taken into account because injections on an as-needed basis would have a lower risk of serious systemic events than scheduled monthly injections.[15], [17] This lower risk of ischaemic events that coincides with a lower versus higher dosage of bevacizumab has been documented in cancer patients by one recently published meta-analysis.[42] Our meta-analysis also showed that death from vascular causes, death from all causes, and nonfatal or serious adverse ocular events were favourable in the ranibizumab treatment group, whereas nonfatal arterial thrombotic events favoured the ranibizumab treatment group. However, no statistically significant differences for the four adverse event endpoints were found between the two arms.

The strengths of this meta-analysis include the comprehensive literature review, strict inclusion criteria, large number of patients analysed, inclusion of the most up-to-date published RCT trial data, and robustness of the findings. By examining both the visual efficacy acuity and serious adverse events, the potential utility of bevacizumab instead of ranibizumab can be adequately evaluated. The present meta-analysis also resolved the question of the inadequate power of individual studies to compare the differences in harm between the two treatments. For example, only four studies included in this review reported any data on death from all causes for more than 100 patients.[14]–[17] A major strength of the current meta-analysis is that the pooled results allowed for the examination of both bevacizumab and ranibizumab for potential ophthalmic diseases related to neovascularisation.

There are also limitations that should be noted regarding this analysis. The first possible limitation of this study is the heterogeneity of the studies regarding different ophthalmic diseases, including AMD, DME, PM, and RAP, which were assumed to have equivalent therapeutic responses for anti-VEGF treatment due to the active role of neovascularisation in their pathophysiology. Furthermore, the paucity of RCT trials comparing ranibizumab and bevacizumab in patients with non-AMD ophthalmic diseases was investigated; the non-inferiority and safety profile between bevacizumab and ranibizumab in other diseases still needs to be further elucidated. The second limitation is the lack of individual-level data, which prohibited the evaluation of the associations between individual variables with study outcomes. Instead, we used between-study meta-regressions when possible. Third, our results are limited to Western populations due to the absence of the data from Eastern populations. Fourth, due to the absences of the detailed definition of serious adverse events for each trial, we only used the adverse information judged by investigators, such as nonfatal or fatal thrombotic events. Therefore, information bias associated with the different definitions could not be excluded, if any was present. Fifth, small sample size of the trial included in the current analysis might have potential impact on detecting the rare adverse events, the bias should be taken into account. Sixth, this meta-analysis only included serious adverse events that had a potential relationship with anti-VEGF therapies.[37], [38] Finally, all studies included in this meta-analysis had a short-term follow-up of less than 2 years, and five studies had a sample size of less than 100 participants. Further larger trials with long-term outcome data are needed.

In summary, ranibizumab treatment resulted in slightly more visual acuity improvement than did bevacizumab, but there were no significant differences. Safety profiles, especially regarding serious systemic events, were also more favourable for ranibizumab than for bevacizumab. The two anti-VEGF agents and their treatment patterns should be carefully chosen by weighing the costs and health benefits. Further studies are needed to compare the effective and safety outcomes between bevacizumab and ranibizumab for ophthalmic diseases, and the appropriate doses of each is an important issue that merits further evaluation by high-quality RCTs. Finally, a high priority should be placed on the need for a detailed description of adverse events, especially serious systemic events, in primary studies.

## Supporting Information

Checklist S1 PRISMA Checklist.
(DOC)
